# Optimizing Welding Sequence and Improving Welding Process for Marine Thick-Walled Circular Pipes

**DOI:** 10.3390/ma18174128

**Published:** 2025-09-02

**Authors:** Tao Ma, Mingguan Fan, Haipeng Miao, Wei Shang, Mingxin Yuan

**Affiliations:** 1 College of Electrical and Information Engineering, Hunan University, Changsha 410082, China; jarimt@163.com; 2 Jiangsu Automation Research Institute, Lianyungang 222000, China; 3 COSCO Shipping Heavy Industry (Yangzhou) Co., Ltd., Yangzhou 225000, China; fan.mingguan@coscoshipping.com (M.F.); shang.wei@coscoshipping.com (W.S.); 4 School of Mechanical Engineering, Jiangsu University of Science and Technology, Zhenjiang 212100, China; 231210201119@stu.just.edu.cn

**Keywords:** thick-walled circular pipe welding, welding sequence optimization, welding process improvement, multi-layer multi-pass welding, welding simulation

## Abstract

To reduce welding deformation during the automated welding of thick-walled pipes in shipbuilding and thereby improve welding quality, a segmented multi-layer multi-pass welding sequence optimization and process improvement strategy is proposed. Firstly, based on a welding model for thick-walled pipes, a multi-layer multi-pass welding trajectory equation is established. A double-ellipsoidal moving heat source is adopted to design a circular multi-layer multi-pass double-ellipsoidal heat source model. Secondly, three circular pipe workpieces with different wall thicknesses are selected, and four segmented welding sequences are simulated using welding finite element analysis (FEA). Finally, based on the optimal segmented welding sequence, the welding process is improved, and optimal welding process parameters are determined based on deformation and residual stress analysis. The results of the segmented multi-layer multi-pass welding sequence optimization show that the skip-symmetric welding method yields the best results for thick-walled circular pipes. Compared to other welding sequences, it reduces welding deformation by an average of 6.50% and welding stress by an average of 5.37%. In addition, process improvement tests under the optimal welding sequence indicate that the best welding quality is achieved under the following conditions: for 10 mm thick pipes—200 A current, 24 V voltage, and 11.5 mm/s welding speed; for 15 mm thick pipes—215 A, 24.6 V, and 10 mm/s; and for 20 mm thick pipes—225 A, 25 V, and 11 mm/s.

## 1. Introduction

Welding is a primary manufacturing technique in shipbuilding, particularly in the assembly of medium and small ship structures, where large-diameter thick-walled circular pipes—often exceeding 50 cm in diameter—are commonly used. These pipes play critical roles in various shipboard systems such as ballast water, fuel delivery, cargo transport, and cooling and firefighting systems. As such, they not only require excellent mechanical properties and corrosion resistance but must also maintain long-term structural stability and safety under complex operating conditions onboard ships [1,2]. Due to significant temperature fluctuations during welding, large-diameter thick-walled pipes are prone to thermal cracking. Moreover, localized thermal expansion and contraction can lead to considerable deformation and residual stress, adversely affecting weld quality [3]. Therefore, selecting an appropriate welding sequence and optimal welding parameters is essential to effectively suppress such issues. At present, most large-diameter thick-walled circular pipe welding tasks in shipbuilding are still performed manually. Robotic automated welding requires the use of positioners to carry out the process. However, due to the structural characteristics of large diameters and thick walls, the choice of welding sequence and process during segmented welding directly impacts weld quality, and improper selection can lead to severe consequences such as pipe leakage [4]. As a result, optimizing the welding sequence and process for large-diameter thick-walled circular pipes has become a key research focus in the field of robotic automated welding [5].

Yang et al. [6] conducted two welding sequence experiments on car body structures and identified the optimal sequence based on three criteria: quality, efficiency, and cost. However, as only two welding sequences were tested, the optimal sequence could not be conclusively determined. Huo et al. [7] also optimized the welding sequence of cylindrical structures through experiments, but the scope was limited and focused on thin-walled, small-diameter elliptical cylinders. With advancements in computer technology, simulation-based welding analysis for circular pipes has rapidly progressed. Luo et al. [8] simulated welding of X80 pipelines to evaluate fusion defects, thus avoiding repeated on-site experimental trials. Li et al. [9] analyzed the effects of welding processes on residual stress in X80 pipelines through simulation, and further enhanced joint durability by improving the welding procedure. However, such steels are rarely used in the shipbuilding industry. Xie et al. [10] used ANSYS to simulate welding sequences for medium-thickness flat plates and identified optimal sequences based on residual stress distribution. Nevertheless, these sequences are not suitable for circular pipes with circumferential welds. Yin et al. [11] designed a pipe welding filling strategy, but it applies only to narrow welds with multi-layer single-pass configurations. Wang et al. [12] simulated two welding sequences for X80 pipelines and identified the optimal one; however, due to the thin wall thickness of the pipes, the findings may not be applicable to thick-walled marine pipes. Mi et al. [13] conducted thermal and stress field simulations of canopy circumferential welds, but the study focused on single-pass long circumferential welds, which are also not applicable to the thick-walled circular pipe welds discussed herein. Gao et al. [14] simulated welding and optimized the sequence for thin-walled composite metal pipes with multi-layer single-pass processes, which differ from the automated multi-layer multi-pass welding of marine thick-walled circular pipes presented in this study. Ren et al. [15] performed welding sequence simulations for long-distance oil and gas pipelines and determined the optimal sequence based on deformation and residual stress. However, the study did not address segmented welding sequence optimization specific to large-diameter pipe structures.

From the existing literature, it is evident that current welding sequence optimization studies primarily focus on thick flat plate welds or multi-layer single-pass circumferential welds. In contrast, marine pipelines often require large-diameter, thick-walled circular pipes capable of withstanding compressive and bending loads, which must be welded using multi-layer multi-pass processes. Therefore, this study first establishes a multi-layer multi-pass welding trajectory equation for thick-walled circular pipes and designs a corresponding double-ellipsoidal moving heat source. Subsequently, welding sequence optimization is performed for pipes with varying wall thicknesses. Finally, based on the optimal welding sequence, process improvements are proposed for different wall thicknesses.

## 2. Welding Model and Trajectory Design

### 2.1. Welding Model Establishment

**Figure 1:**
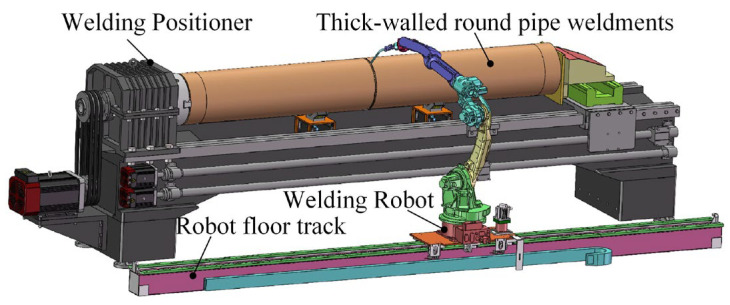
Construction of a welding platform for thick-walled circular pipes for ships.

To address the challenges of automated welding for ballast and fuel delivery pipelines in ships, a robotic welding platform for thick-walled circular pipes was established, as shown in Figure 1. In the setup, the circular pipe workpiece is mounted on a positioner, while the welding robot is installed on a floor-mounted rail system. By coordinating with the positioner, the robot enables segmented welding of the thick-walled circular pipe.

**Figure 2:**
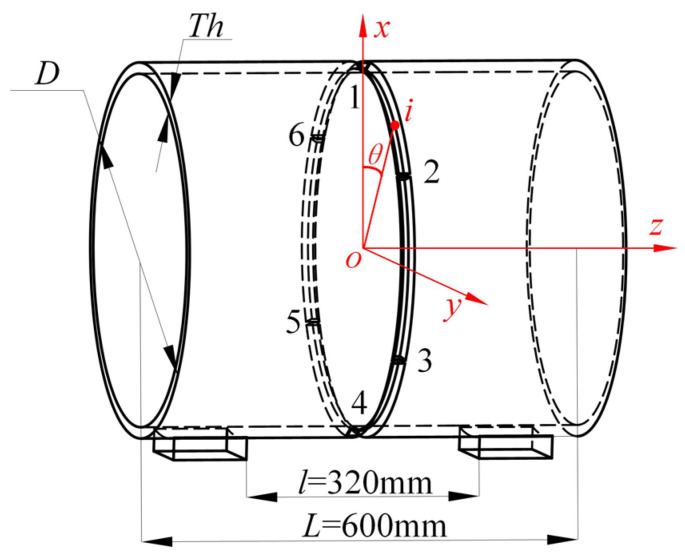
Simplified Welding Model.

In the welding of thick-walled circular pipes, six evenly spaced tack welds are first applied manually to fix the workpiece, with each weld having a width of no less than 15 mm. This is followed by manual gas tungsten arc welding (GTAW) for root pass welding. Subsequently, segmented automated continuous welding is performed using a robotic system. In actual welding operations, the positioner rotates the workpiece to coordinate with the robot for automated welding. However, this rotation is difficult to replicate in welding simulations. Therefore, to simplify the model, the thick-walled circular pipe is placed horizontally on two support blocks, and a coordinate system is established as shown in Figure 2. A moving heat source is used for welding simulation, in which the optimal welding sequence is first identified [16], followed by welding process improvements to ensure optimal weld quality.

In the figure, *D* denotes the diameter of the thick-walled circular pipe, *Th* represents the wall thickness, *L* is the length of the pipe, and *l* is the spacing between the support blocks.

### 2.2. Welding Trajectory Design

**Figure 3:**
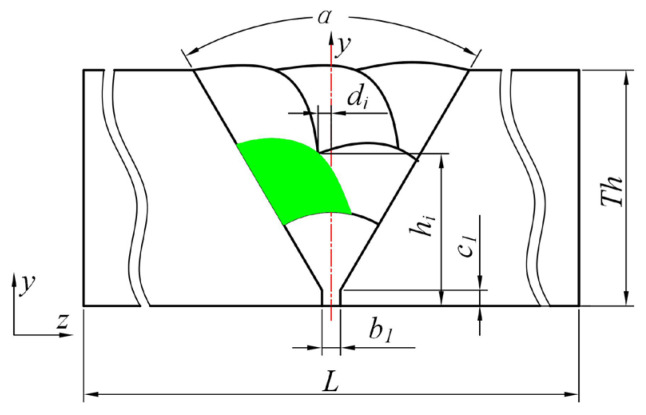
Schematic diagram of a thick-walled circular pipe weld seam.

As previously mentioned, the root pass of the first weld bead on the thick-walled circular pipe is performed manually using gas tungsten arc welding (GTAW), while the subsequent weld beads are completed by a robot employing CO_2_ gas shielded arc welding. The weld seam is a multi-layer, multi-pass circumferential weld. According to the equal-height and equal-area method, the weld shape of the thick-walled circular pipe is designed as shown in Figure 3 [17]. The green portion in the figure represents the schematic diagram of the *i*-th weld.

In the figure, *α* represents the groove angle of the weld seam, *b*_1_ denotes the groove root gap, *c*_1_ is the groove root face (land), *d_i_* is the distance from the welding torch to the *xy* plane in weld pass *i*, and *h*_i_ is the distance from the welding torch to the inner wall of the thick-walled circular pipe in weld pass *i*.

The weld groove angle *α*, root face *c*_1_, and gap *b*_1_ are selected based on the workpiece dimensions and industry standards. When the workpiece has no groove preparation, the parameter variable *θ* is introduced, where *θ* is the angle between the projection of any weld pass *i* on the weld seam onto the *xoy* plane and the positive *x* direction. Using the equal-height and equal-area method, the welding torch trajectory for each weld pass can be obtained as follows:

(1) $$\left\{\begin{array}{l} x=(\frac{D}{2}-(Th-h_{i}))\cos\theta \\ y=(\frac{D}{2}-(Th-h_{i}))\sin\theta ,\theta \in (0,2\pi )\\ z=d_{i} \end{array}\right.$$

Let *r_i_* = *D*/2 − (*Th* − *h_i_*), then the trajectory equation for each weld pass is:

(2) $$\left\{\begin{array}{l} x=r_{i}\cos\theta \\ y=r_{i}\sin\theta \\ z=d_{i} \end{array}\right.,\theta \in (0,2\pi )$$

## 3. Heat Source Model Establishment

**Figure 4:**
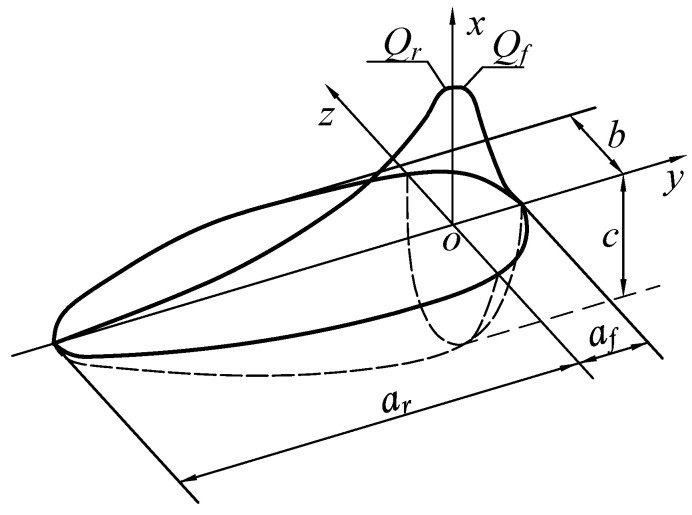
Double ellipsoid heat source model.

In thick-walled pipe welding simulations, most researchers have found that the double-ellipsoidal heat source model better approximates actual welding processes [18,19] and yields more accurate numerical results. Based on the aforementioned workpiece coordinate system (*x*, *y*, *z*), the double-ellipsoidal heat source model *q*(*x*, *y*, *z*) is established as shown in Figure 4.

The heat source expression for the front half-ellipsoid is:

(3) $$q_{f}(x,y,z)=\frac{6\sqrt{3}f_{1}Q_{f}}{\pi a_{f}bc\sqrt{\pi }}\exp\left\{-3\left[{\left(\frac{x}{c}\right)}^{2}+{\left(\frac{y}{a_{f}}\right)}^{2}+{\left(\frac{z}{b}\right)}^{2}\right]\right\}$$

The heat source expression for the rear half-ellipsoid is:

(4) $$q_{r}(x,y,z)=\frac{6\sqrt{3}f_{2}Q_{r}}{\pi a_{r}bc\sqrt{\pi }}\exp\left\{-3\left[{\left(\frac{x}{c}\right)}^{2}+{\left(\frac{y}{a_{r}}\right)}^{2}+{\left(\frac{z}{b}\right)}^{2}\right]\right\}$$

where *Q_f_* and *Q_r_* represent the instantaneous heat input of the front and rear half-ellipsoids, respectively; *a_f_* and *a_r_* denote the semi-axis depths along the *y*-axis for the front and rear half-ellipsoids; *b* and *c* are the semi-axis depths along the *z*-axis and *x*-axis, respectively; *f*_1_ and *f*_2_ are the energy distribution coefficients for the front and rear half-ellipsoids, which satisfy the condition:

(5) $$f_{1}+f_{2}=2$$

**Figure 5:**
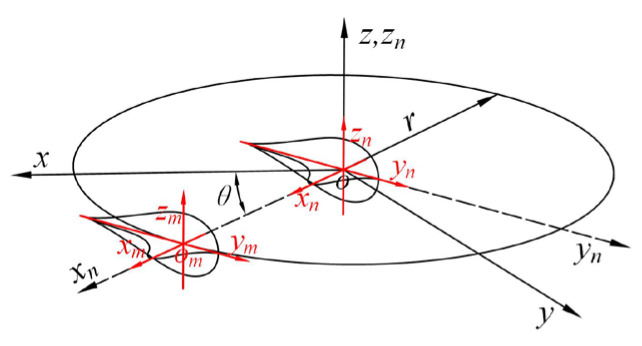
Double ellipsoid heat source trajectory diagram.

During welding simulation, the heat source moves with the welding torch. However, the double-ellipsoidal moving heat source is predominantly applied to linear weld seams. To address the challenge of applying the double-ellipsoidal heat source along a circumferential trajectory, this study proposes a coordinate rotation method, as illustrated in Figure 5.

The double-ellipsoidal heat source *q* (*x*, *y*, *z*) is rotated around the *z*-axis by an angle *θ*, resulting in the heat source trajectory coordinates *q* (*x_n_*, *y_n_*, *z_n_*) as shown in the following equation.

(6) $$\left[\begin{array}{l} x_{n}\\ y_{n}\\ z_{n} \end{array}\right]=\left[\begin{array}{ccc} \cos\theta & -\sin\theta & 0\\ \sin\theta & \cos\theta & 0\\ 0 & 0 & 1 \end{array}\right]\left[\begin{array}{l} x\\ y\\ z \end{array}\right]$$

Introducing the angular velocity *w* and time *t*, and letting *θ* = *wt*, the expression can be further simplified as:

(7) $$\left\{\begin{array}{l} x_{n}=x\cos wt-y\sin wt\\ y_{n}=x\sin wt+y\cos wt\\ z_{n}=z \end{array}\right.$$

Furthermore, since the double-ellipsoidal ring heat source rotates along a circular weld seam with radius *r_i_* in the *x_n_* direction, the heat source trajectory equation *q*(*x_m_*, *y_m_*, *z_m_*) can be derived by combining with Equation (2) as follows:

(8) $$\left\{\begin{array}{l} x_{m}=x\cos wt-y\sin wt-r_{i}\\ y_{m}=x\sin wt+y\cos wt\\ z_{m}=z-d_{i} \end{array}\right.$$

By combining Equations (3) and (4), the front half-ellipsoid moving heat source equation *q_f_* (*x*, *y*, *z*) at time *t* can be expressed as:

(9) $$q_{f}(x,y,z,t)=\frac{6\sqrt{3}f_{1}Q_{f}}{\pi a_{f}bc\sqrt{\pi }}\exp\left\{-3\left[{\left(\frac{x\cos wt-y\sin wt-r_{i}}{c}\right)}^{2}+{\left(\frac{x\sin wt+y\cos wt}{a_{f}}\right)}^{2}+{\left(\frac{z-d_{i}}{b}\right)}^{2}\right]\right\}$$

Similarly, the rear half-ellipsoid moving heat source equation *q_r_* (*x*, *y*, *z*) at time *t* is given by:

(10) $$q_{r}(x,y,z,t)=\frac{6\sqrt{3}f_{2}Q_{r}}{\pi a_{r}bc\sqrt{\pi }}\exp\left\{-3\left[{\left(\frac{x\cos wt-y\sin wt-r_{i}}{c}\right)}^{2}+{\left(\frac{x\sin wt+y\cos wt}{a_{r}}\right)}^{2}+{\left(\frac{z-d_{i}}{b}\right)}^{2}\right]\right\}$$

Since this moving heat source follows a circular trajectory with radius *r_i_*, the welding speed *v*(*w*) is given by:

(11) $$v(w)=r_{i}w$$

To simulate realistic welding conditions, the angular velocity *w* can be derived inversely from the welding speed *v*(*w*), and then substituted into Equations (9) and (10) to approximate the double-ellipsoidal moving heat source corresponding to actual operating conditions.

## 4. Welding Sequence Optimization

To determine the optimal welding sequence for thick-walled circular pipes, welding simulations were conducted using ANSYS Workbench. First, a simplified model of the thick-walled circular pipe and the corresponding weld geometry was created in SolidWorks (2020). Then, based on the equal-height and equal-area method, the values of *r*_*i*_ and *d*_*j*_ were calculated, and the moving heat source trajectory equations for each weld pass were established. Subsequently, the weld model was imported into ANSYS Workbench, where nonlinear material properties were defined. The transient thermal field was first computed and then imported into the transient structural module to calculate residual stress and deformation. Finally, the simulation results were compared to determine the optimal welding sequence.

### 4.1. Pre-Simulation Processing

To enhance the generality of the study on segmented multi-layer multi-pass welding of thick-walled circular pipes, three pipe specimens with wall thicknesses *Th* of 10 mm, 15 mm, and 20 mm were selected for welding simulation. The remaining parameters of the welding model are listed in Table 1.

**Figure 6:**
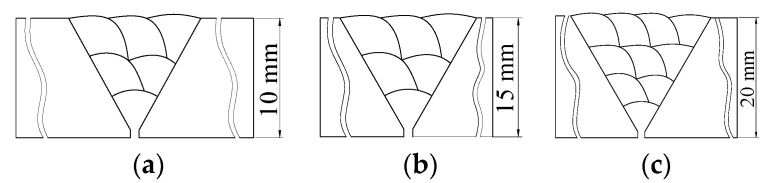
Weld shapes of circular pipes with different wall thicknesses: (**a**) 10 mm wall thickness; (**b**) 15 mm wall thickness; (**c**) 20 mm wall thickness.

In practical welding applications, circular pipes with a diameter greater than 8 mm typically require three or more weld layers. Since the 10 mm wall thickness is relatively thin, the welding voltage and current used for 10 mm thick pipes are lower than those for 15 mm and 20 mm pipes when the welding speed remains constant. As a result, the weld cross-section is smaller. The weld geometries for the three wall thicknesses are shown in Figure 6.

For 10 mm and 15 mm thick pipes, the weld consists of three layers with six passes, while for the 20 mm thick pipe, the weld consists of four layers with ten passes.

**Figure 7:**
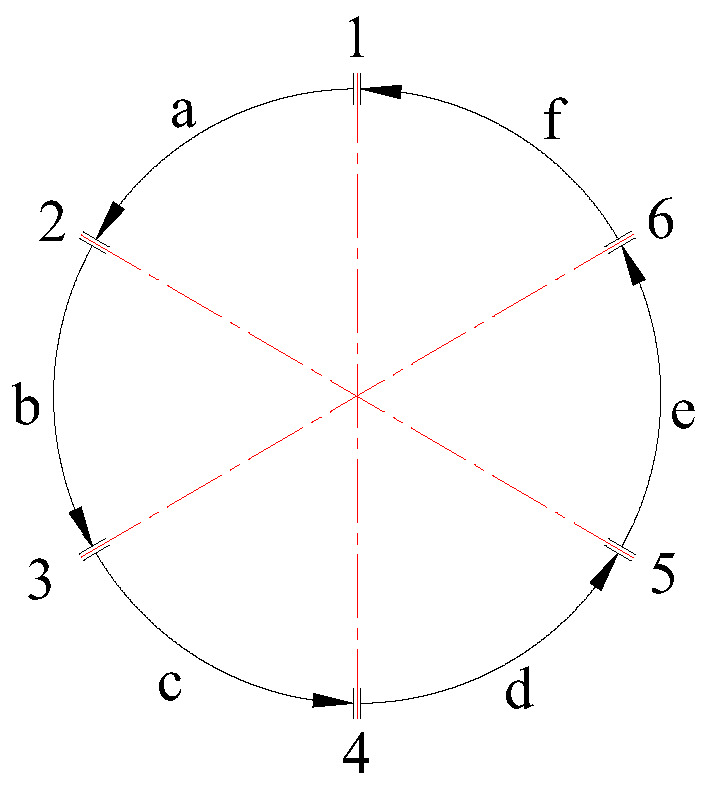
Segmented Welding Method for Thick-walled Circular Pipe.

Prior to performing automated robotic welding on the circular pipe, six tack welds were manually applied to secure the workpiece. As a result, the circular weld seam was divided into six segments, as illustrated in Figure 7. In the figure, numerals 1 to 6 represent the six tack welds, while letters a to f denote the six corresponding welding segments.

**Figure 8:**
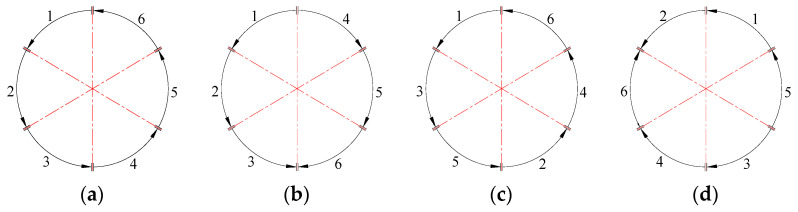
Sectional Welding Sequence for Thick-walled Circular Pipes: (**a**) N1; (**b**) N2; (**c**) N3; (**d**) N4.

During the welding process, abrupt thermal expansion and contraction of the workpiece induce stress and strain, which in turn lead to welding deformation and residual stress. To reduce welding deformation in thick-walled circular pipes, four welding sequence strategies, as illustrated in Figure 8, are proposed in this study. The numbers 1–6 in the diagram indicate the sequential order of welding.

N1: Sequential Circulation Method—welds are completed sequentially in a single rotational direction.

N2: Symmetric Continuous Method—the weld seam is divided into two large sections (left and right); three segments are welded continuously in one direction, then the remaining three are welded in the reverse direction, starting from the initial point.

N3: Jump-Symmetric Method—welding is performed in a continuous, symmetric, and alternating pattern following a predefined direction.

N4: Cross-Symmetric Method—the weld seam is divided into four parts; two larger sections (top and bottom) are welded first in a crosswise manner, followed by the remaining two smaller sections (left and right).

After constructing the welding model, it was imported into ANSYS Workbench, and material properties were assigned. As marine thick-walled circular pipes are often used in oceanic and harsh climatic environments, they must possess high impact resistance and fatigue strength to withstand wave-induced loads, as well as long-term durability under low temperatures and cold climates. Therefore, the material must also be capable of resisting high humidity and salt spray corrosion.

Based on shipbuilding industry standards [20] and practical applications in shipyards, this study selected Q460 high-strength structural steel, which meets the complex requirements of both nearshore and ocean-going vessels, as well as offshore engineering structures. According to the standards for seamless carbon steel pipes used in shipbuilding, the chemical composition of Q460 steel is listed in Table 2.

During the welding process, the temperature of the workpiece material rises instantaneously under the action of the heat source, causing it to melt and form a molten pool, which is then filled with filler wire to create the weld seam. In this process, the material properties of the workpiece undergo nonlinear changes.

**Figure 9:**
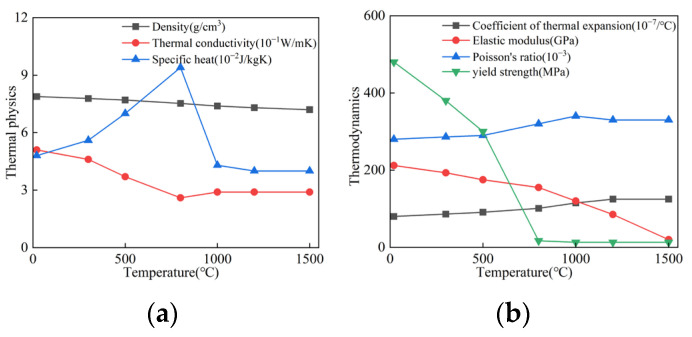
Nonlinear high-temperature properties of Q460 steel: (**a**) Thermal-physical properties of the steel; (**b**) Thermo-mechanical properties of the steel.

The calculation of the welding temperature field depends on the thermal-physical properties of the steel, while welding deformation and residual stress are influenced by its thermo-mechanical properties. Based on the relevant literature [21], the nonlinear thermal-physical and thermo-mechanical properties of Q460 high-strength structural steel were obtained and are shown in Figure 9.

After assigning the material properties to the circular pipe in the ANSYS (2024R1) Workbench, mesh generation must be performed. The mesh size directly affects the computational accuracy. An excessively fine mesh can waste computational resources and reduce simulation efficiency.

**Figure 10:**
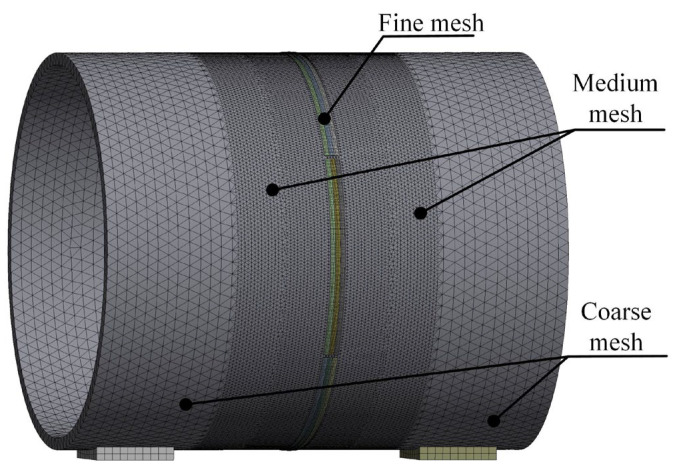
Mesh division of the thick-walled circular pipe.

Therefore, in this study, a multi-scale meshing strategy was adopted: a coarse mesh was applied to regions far from the weld seam, a moderately fine mesh was used near the weld seam, and a fine mesh was applied to the weld seam and its surrounding area. The weld seam was meshed using multi-region hexahedral elements of type SOLID186, while the remaining parts of the thick-walled circular pipe were automatically meshed using SOLID187 elements, as shown in Figure 10.

### 4.2. Simulation Method

After completing the pre-processing setup, numerical simulation of the thick-walled circular pipe welding was performed. The first step is to calculate the temperature field during the welding process of the thick-walled circular pipe. In finite element theory, the transient heat conduction matrix for the thick-walled circular pipe is given by:

(12) $$\left[C\right]\left\{\dot{T}\right\}+\left[K\right]\left\{T\right\}=\left\{Q\right\}$$

where [*K*] is the total heat conduction matrix of the thick-walled circular pipe element, [*Q*] is the total heat load matrix representing the heat source, [*C*] is the specific heat capacity matrix, {*T*} denotes the nodal temperature vector of the thick-walled circular pipe, and {$\dot{T}$} represents the time derivative of the nodal temperature vector.

Due to the localized rapid temperature rise and subsequent sudden cooling caused by the welding heat source on the pipe, and the nonlinear variation of material physical properties with temperature during the entire process, the above equation can be expressed as:

(13) $$\left[C(T)\right]\left\{\dot{T}\right\}+\left[K(T)\right]\left\{T\right\}=\left\{Q(T)\right\}$$

Under the heating of the heat source, the thick-walled circular pipe workpiece undergoes thermal expansion and contraction. However, due to constraints imposed on each micro-element of the pipe by various factors, free expansion or contraction is restricted, resulting in thermal stresses.

According to thermoelasticity theory, the total elastic strain *d*{*ε*}*_all_* of the pipe material is assumed to be composed of the sum of the elastic strain increment *d*{*ε*}*_el_* caused by external forces and the thermal strain increment *d*{*ε*}*_hot_* caused by temperature changes. The elastic strain {*ε*}*_el_* is calculated based on elastic mechanics principles, while the thermal strain {*ε*}*_hot_* is computed according to thermoelasticity theory.

(14) $$d{\left\{\varepsilon \right\}}_{all}=d{\left\{\varepsilon \right\}}_{el}+d{\left\{\varepsilon \right\}}_{hot}$$

Considering that plastic deformation occurs in the vicinity of the weld pool during the welding process of the circular pipe workpiece [22,23], the strain equation representing the total strain increment {*ε*}*_all_* can be expressed as:

(15) $$d{\left\{\varepsilon \right\}}_{all}=d{\left\{\varepsilon \right\}}_{el}+d{\left\{\varepsilon \right\}}_{p}+d{\left\{\varepsilon \right\}}_{hot}$$

where, *d*{*ε*}_*p*_ represents the plastic strain increment, which is related to the yield function of the circular pipe material.

The relationship between the nodal displacement increment {*δ*}_*e*_ of element *e* in the circular pipe workpiece and the initial strain increment [*R*]^*e*^ induced by temperature can be expressed as:

(16) $$\left\{R^{e}\right\}=\left[K^{e}\right]\left\{\delta^{e}\right\}$$

(17) $${\left[R\right]}^{e}=\int {\left[B\right]}^{T}\left[C\right]dVdT$$

(18) $${\left[K\right]}^{e}=\int {\left[B\right]}^{T}\left[D\right]\left[B\right]dV$$

where [*K*]^*e*^ is the element stiffness matrix, and [*B*] is the coefficient matrix related to the element nodes.

During the welding of thick-walled circular pipes, complex welding conditions may lead to certain deviations. To minimize these discrepancies, a correction coefficient matrix [*G*] is introduced in this study to adjust the stiffness matrix. The corrected element stiffness matrix is expressed as follows:

(19) $${\left[\bar{K}\right]}^{e}=\int {\left[B\right]}^{T}\left[D\right]\left[B\right]\left[G\right]dV$$

The nodal displacement increment {*δ*}_*e*_ of the thick-walled circular pipe can be expressed as:

(20) $$\left\{\delta^{e}\right\}={\left[\int {\left[B\right]}^{T}\left[D\right]\left[B\right]\left[G\right]dV\right]}^{-1}\int {\left[B\right]}^{T}\left[C\right]dVdT$$

The total welding deformation *w* of the circular pipe is given by:

(21) $$w=\sum_{i=1}^{\Omega }I_{e}{\left\{\delta \right\}}^{e}$$

Based on finite element theory, this study simulated the temperature fields of thick-walled circular pipes under different welding sequences using Ansys (2024R1) Workbench Mechanical’s Transient Thermal module (Software source: Ansys, Inc., Canonsburg, PA, USA).

The simulations were conducted on a computer equipped with an Intel (Intel Corporation, Santa Clara, CA, USA) i7-11700K processor, 96 GB of RAM, and a GP100 graphics card. The resulting temperature field data were then imported into the transient structural module to calculate the deformation and stress corresponding to each welding sequence.

In actual welding processes, since thick-walled circular pipes are typically welded directly in shipyards where the environment is relatively enclosed, the convective heat transfer between the workpiece surface and air was set to 10 W/(m^2^·°C) when defining the boundary conditions of the temperature field. As the heat source in this study adopts a moving double-ellipsoidal model, the heat source was applied in the Workbench transient thermal module via APDL command streams. To reduce computation time, the simulation time step was set to 1 s, and the transient integration option was enabled. To better approximate the actual welding process, the element birth and death technique in ANSYS was employed.

In this sequential coupled thermal-mechanical analysis, the temperature field data from the thermal analysis based on SOLID70 elements is automatically mapped to the SOLID186/SOLID187 structural model using ANSYS’s LDREAD command. This process employs a shape function interpolation algorithm to automatically generate temperature values for the intermediate nodes of quadratic elements, enabling precise transfer of thermal-mechanical loads from linear elements to quadratic elements. This process automatically interpolates nodal temperatures from the linear thermal mesh onto the quadratic structural mesh, ensuring consistency of the coupled thermo-mechanical model. Sequential coupling of transient thermal analysis and transient structural thermomechanical analysis was performed without considering the effects of metallurgical phase transformations. The mechanical constitutive model adopted a bilinear isotropic hardening rule based on the von Mises yield criterion. Since welding simulation involves large deformation problems, the “Full Newton-Raphson” method was selected in the transient structural module, with Weak Spring and Large Deflection options enabled. To improve computational efficiency, automatic line search and the SPARSE solver were used. Fixed supports were applied at the bottom of the support blocks, and gravity G was applied in the negative *x*-direction.

In the welding sequence optimization simulations, the 10 mm thick pipe used a welding current of 190 A, a voltage of 23.6 V, and a welding speed of 10.5 mm/s. The 15 mm and 20 mm thick pipes used a welding current of 220 A, a voltage of 24.8 V, and a welding speed of 10.5 mm/s. According to the actual working conditions of the shipyard, the welding thermal efficiency is 0.7. The parameters of the double ellipsoidal heat source are shown in Table 3.

For each wall thickness, different welding sequences were compared in terms of welding deformation and residual stress to determine the optimal welding sequence. Excluding the first manually applied TIG root pass, the 10 mm and 15 mm pipes required five additional weld passes, completing welding at 750 s. The 20 mm pipe required nine additional weld passes, with welding completed at 1350 s.

### 4.3. Results and Discussion

**Figure 11:**
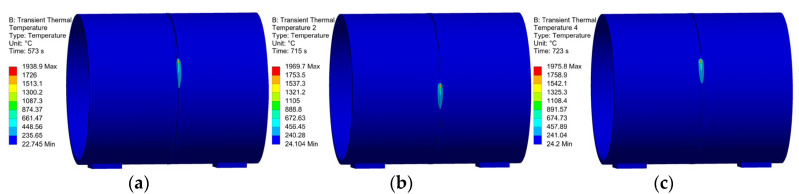
Welding heat source loading: (**a**) 573 s heat source simulation; (**b**) 715 s heat source simulation; (**c**) 723 s heat source simulation.

In welding simulation, strict requirements are imposed on the heat input. After applying the heat source, the heat source distribution is shown in Figure 11. It can be observed from the figure that the heat source is successfully applied to the circular pipe weld seam and moves rotationally along the weld seam, ensuring the accuracy of the heat source application and thus guaranteeing the correctness of the welding simulation.

**Figure 12:**
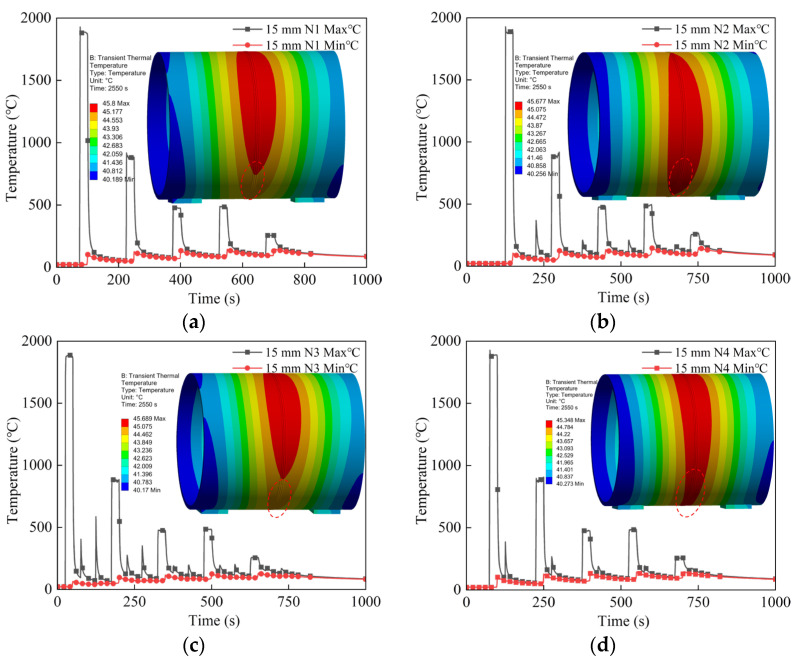
Temperature monitoring of d-section weld with a wall thickness of 15 mm under four welding sequences: (**a**) N1; (**b**) N2; (**c**) N3; (**d**) N4.

Taking the 15 mm wall thickness circular pipe as an example, after the heat source is applied, the temperature variation of segment *d* in the first weld pass was monitored under four different welding sequences during robotic welding. As shown in Figure 12, the temperature of segment *d* rapidly rises above the steel melting point during welding and sharply drops after welding completion.

With the addition of subsequent weld passes, previously deposited weld beads are reheated by the existing molten pool, causing temperature increases. Moreover, the higher the number of weld layers, the less influence the current molten pool has on the underlying welds [24]. Slight differences in the temperature variations of segment *d* under different welding sequences result in localized uneven heating and cooling, which in turn easily cause welding deformation and residual stress.

Fifteen minutes after welding completion, the overall temperature of the pipe for all four welding sequences stabilized around the mid-40s °C, with the maximum temperature dropping to approximately 45 °C. This is very close to actual welding conditions in shipyards, thereby validating the accuracy of the welding temperature field simulated in this study.

**Figure 13:**
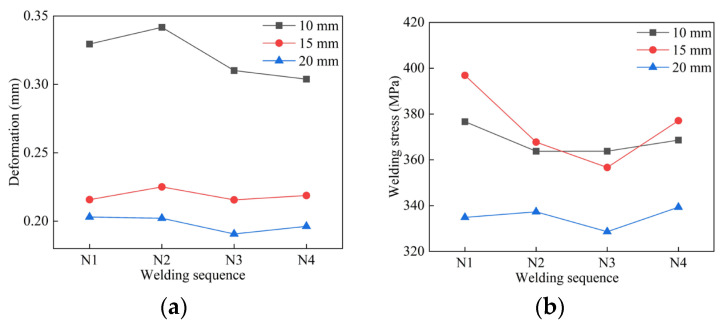
Welding results of different welding sequences: (**a**) Segmented welding deformation results; (**b**) Segmented welding stress results.

After calculating the temperature field in the transient thermal analysis, the data were imported into the transient structural module, and APDL scripts were written to compute welding deformation and residual stress for pipes with three different wall thicknesses under various welding sequences. The simulation results are shown in Figure 13. For the 10 mm thick pipe, the welding deformation under the N3 sequence was 0.3101 mm, and the post-weld residual stress was 363.78 MPa. Compared to the maximum welding deformation of 0.34172 mm caused by N2, this represents a reduction of 9.25%, and compared to the maximum welding residual stress of 376.67 MPa caused by N1, a reduction of 3.42%.

For the 15 mm thick pipe, the welding deformation under N3 was 0.21562 mm, and the residual stress was 356.67 MPa. Compared to the maximum welding deformation of 0.22507 mm caused by N2, the deformation decreased by 4.19%, and the residual stress decreased by 10.13% relative to the maximum residual stress of 396.88 MPa caused by N1.

For the 20 mm thick pipe, the welding deformation under N3 was 0.19069 mm, and the residual stress was 328.62 MPa. Compared to the maximum welding deformation of 0.20303 mm caused by N1, the deformation decreased by 6.07%, and the residual stress decreased by 2.57% relative to the maximum residual stress of 337.31 MPa caused by N4.

In summary, the average welding deformation of the three pipe thicknesses under the N3 welding sequence decreased by 6.50%, and the residual stress decreased by 5.37%, indicating that the N3 welding sequence is the optimal choice.

**Figure 14:**
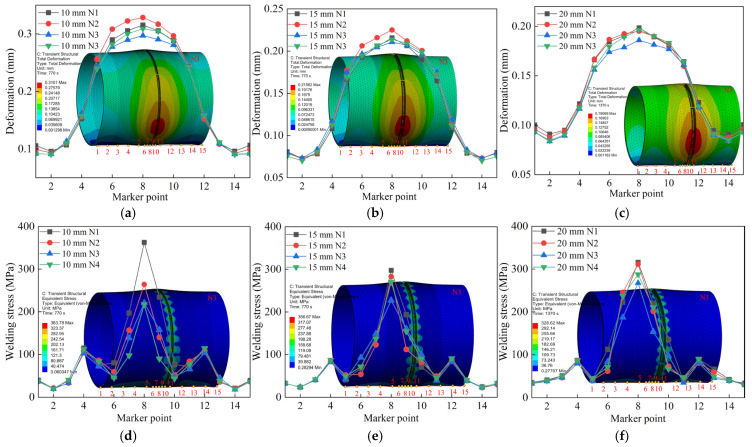
Comparison of axial welding quality of three types of wall thickness circular pipes under different welding sequences: (**a**) Axial deformation of 10 mm segment; (**b**) Axial deformation of 15 mm segment; (**c**) Axial deformation of 20 mm segment; (**d**) Axial stress of 10 mm segment; (**e**) Axial stress of 15 mm segment; (**f**) Axial stress of 20 mm segment.

To further validate that the N3 jump-symmetric welding sequence is optimal, axial monitoring points were selected at the locations of maximum deformation and maximum stress in the circular pipe welding simulations. In Figure 14, 15 monitoring points were selected along the horizontal axis, as indicated by the numbers 1–15 in the figure. The results are shown in Figure 14a–c. From the axial deformation data, it can be seen that the welding deformation of thick-walled circular pipes is primarily concentrated near the weld seam and diffuses outward, extending all the way to the pipe ends, causing an upward warping tendency at the pipe mouths. Although the welding parameters for the 15 mm and 20 mm thick pipes are the same, the 20 mm thick pipe exhibits larger deformation at the edges and more pronounced warping. Under identical welding processes, greater wall thickness leads to more significant thermal expansion effects. Thicker pipes require absorbing more heat due to their larger volume during welding, resulting in greater temperature gradients and larger volumetric expansion. Figure 14d–f shows that the maximum residual stresses occur near the weld spots aligned with the support blocks. The presence of support blocks induces tensile stress at the bottom during welding. In contrast, thinner pipes dissipate heat more quickly, leading to smaller temperature differences and reduced thermal expansion effects.

The welding simulation results indicate that the greatest deformation occurs near the weld spots, while the welding residual stress mainly concentrates around the weld seams. The maximum residual stress occurs near the weld spots at the support block locations [25]. Therefore, before robotic welding, these weld spots require additional reinforcement and thickening during tack welding to prevent weld spot failure caused by excessive deformation or stress during welding. A comprehensive comparison of axial deformation and stress in pipes of three wall thicknesses under different welding sequences shows that the N3 jump-symmetric welding sequence effectively suppresses both welding deformation and residual stress.

**Figure 15:**
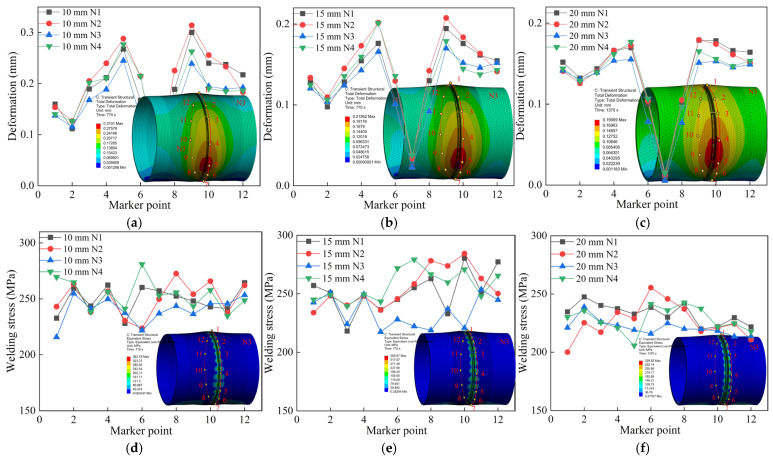
Comparison of radial welding quality of three types of wall thickness circular pipes under different welding sequences: (**a**) Radial deformation of 10 mm segment; (**b**) Radial deformation of 15 mm segment; (**c**) Radial deformation of 20 mm segment; (**d**) Radial stress of 10 mm segment; (**e**) Radial stress of 15 mm segment; (**f**) Radial stress of 20 mm segment.

Furthermore, deformation of the circular pipe was monitored at a distance of 15 mm from the weld seam, and post-weld residual stress was monitored at 5 mm from the weld seam, as shown in Figure 15. Twelve inspection points are uniformly distributed radially along the thick-walled circular pipe, as indicated by numbers 1-12 in the figure. These two regions belong to the welding heat-affected zone (HAZ) [26]. By comparing the radial deformation and stress, it can be seen that the welding performance of the three wall-thickness pipes is optimal under the N3 jump-symmetric welding sequence.

The pipe deformation is minimal at the support blocks and larger on both sides of the bottom, while the residual stress around the weld seam is relatively high. Due to the cyclic reheating caused by multi-layer multi-pass welding in the heat-affected zone, the base metal near this area undergoes thermal expansion and contraction. The closer to the weld zone, the more likely plastic deformation occurs and stress increases [27]. Therefore, welding thick-walled pipes requires not only selecting the optimal welding sequence but also strictly controlling welding parameters during the process. Post-weld annealing treatment and manual correction of deformation areas are also necessary to ensure structural integrity.

To validate the physical behavior predicted by the model, the simulation results for the circular pipe were compared with the existing literature on pipe welding simulations [16,21]. The trends in temperature and stress field distributions align with those reported in the existing literature. This confirms the validity of the present simulation results.

A comprehensive comparison of welding simulations for 10 mm, 15 mm, and 20 mm thick circular pipes under four different welding sequences shows that the N3 jump-symmetric welding sequence yields the best welding performance for thick-walled circular pipes. This welding sequence effectively suppresses welding deformation, reduces post-weld residual stress, and improves overall welding quality.

## 5. Welding Process Improvement

Welding quality is influenced not only by the welding sequence but also by the welding process parameters [28]. Excessively high welding current can cause excessive weld penetration, insufficient weld reinforcement, or burn-through. A high heat input expands the heat-affected zone, leading to degradation of the material’s microstructure and a reduction in overall structural performance [29]. Conversely, insufficient welding current results in inadequate penetration and incomplete fusion of the weld seam [30].

Furthermore, excessively high welding speed tends to cause cracking [31], while too slow a welding speed adversely affects welding efficiency. Therefore, selecting appropriate welding parameters is crucial to effectively improve welding quality. In traditional manual welding of thick-walled circular pipes, welders typically perform welding current tests on scrap steel plates based on their accumulated experience. Then, the initial voltage is determined according to Equation (21), and further fine-tuning is conducted during trial welding until the optimal welding quality is achieved.

However, in robotic automated welding, determining welding parameters through multiple on-site trials is not only inefficient but also requires substantial labor and economic costs. By leveraging empirical data from manual welding combined with software simulation, the trial costs can be significantly reduced, and the efficiency of parameter optimization can be greatly improved.

(22) U=(0.04I+16)±2V

During the welding process, pipes with a wall thickness below 10 mm typically use lower welding currents to avoid full penetration of the pipe wall. Based on practical manual welding experience and combined with Equation (21), the welding process parameters proposed for 10 mm thick pipes are listed in Table 4.

Similarly, the welding process parameters proposed for circular pipes with 15 mm and 20 mm wall thicknesses are presented in Table 5.

**Figure 16:**
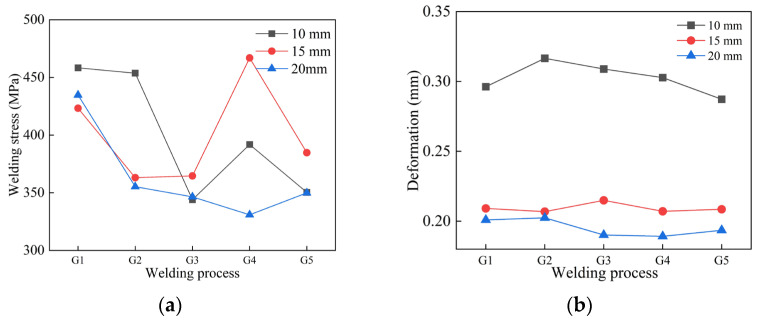
Welding simulation results under different welding processes: (**a**) Welding deformation; (**b**) Welding stress.

After coding the welding parameters into APDL and loading them into Ansys Workbench, welding process simulations for thick-walled circular pipes were conducted based on the N3 jump-symmetric welding sequence. Figure 16 shows the welding deformation and residual stress after simulation. As illustrated, for the 10 mm thick pipe, the G5 welding process produced the smallest deformation of 0.28726 mm. Compared to the maximum deformation of 0.31658 mm, this is a reduction of 0.02932 mm. Regarding residual stress, the welding stress under G3 is similar to that under G5; however, since G5 results in smaller deformation, it is preferred. The welding residual stress for G5 is 350.39 MPa, which is 107.98 MPa less than the maximum stress of 458.37 MPa. For the 15 mm thick pipe, deformation differences among the various welding processes are minor. However, from the residual stress perspective, G2 and G3 have relatively low residual stresses. Since G2 also has smaller welding deformation than G3, G2 is preferred. The welding deformation for G2 is 0.20685 mm, reduced by 0.00806 mm compared to the maximum deformation of 0.21491 mm. The welding residual stress under G2 is 363.1 MPa, which is 103.84 MPa less than the maximum stress of 466.94 MPa.

Similarly, for the 20 mm thick pipe, deformation differences among the welding processes are also small. G3 and G4 cause relatively smaller deformation, but since G4 has lower residual stress, G4 is preferred. The welding deformation under G4 is 0.18919 mm, which is 0.01323 mm less than the maximum deformation of 0.20242 mm. The residual stress under G4 is 346.47 MPa, reduced by 88.27 MPa compared to the maximum stress of 434.74 MPa.

**Figure 17:**
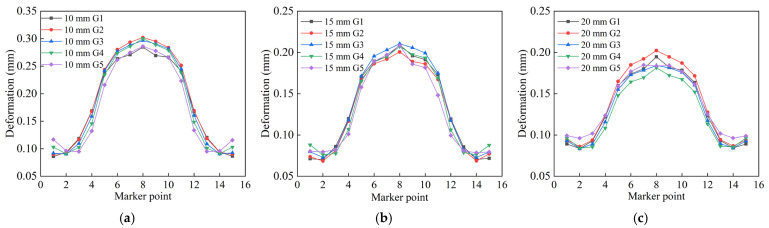

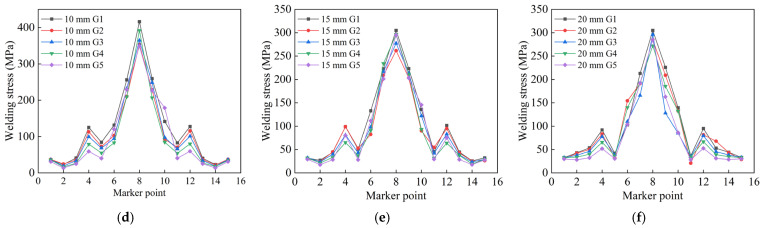
Comparison of axial welding quality of three types of wall thickness circular pipes under different welding processes: (**a**) 10 mm wall thickness axial deformation; (**b**) 15 mm wall thickness axial deformation; (**c**) 20 mm wall thickness axial deformation; (**d**) 10 mm wall thickness axial stress; (**e**) 15 mm wall thickness axial stress; (**f**) 20 mm wall thickness axial stress.

As shown in Figure 17, the axial deformation and axial stress from all welding simulations were compared. Figure 17a,d presents the axial deformation and stress of the 10 mm thick pipe under five welding processes. It can be seen that the axial deformation caused by G1 and G5 processes is similar; however, considering the smaller axial stress generated by G5 in Figure 17d, the G5 welding process is preferred for 10 mm thick pipe welding.

Similarly, for the 15 mm thick pipe, Figure 17b,e shows that both G2 and G5 processes result in relatively small axial deformation. From the axial stress perspective, G2 produces a significantly lower maximum stress than the others, indicating that G2 is the optimal welding process for 15 mm thick pipe welding.

For the 20 mm thick pipe, as illustrated in Figure 17c,f, both G3 and G4 welding processes cause relatively small axial deformation. Combining this with the axial stress results in Figure 17f, G4 also yields lower axial stress. Therefore, the G4 welding process should be selected for welding 20 mm thick pipes.

**Figure 18:**
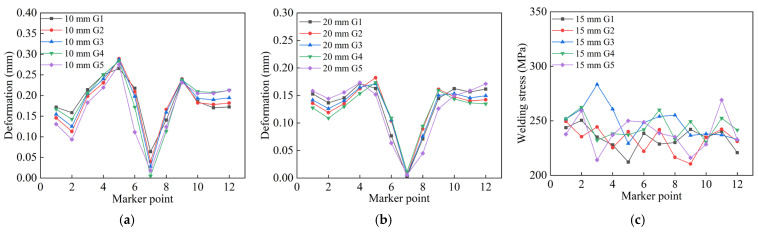

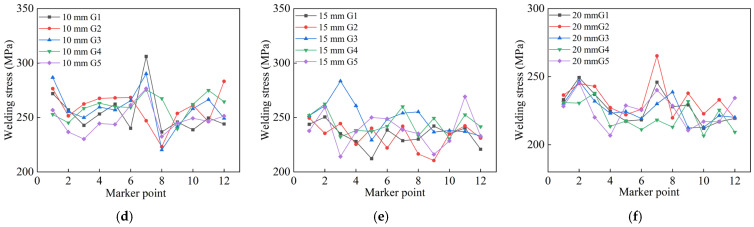
Comparison of radial welding quality of three types of wall thickness circular pipes under different welding processes: (**a**) 10 mm wall thickness radial deformation; (**b**) 15 mm wall thickness radial deformation; (**c**) 20 mm wall thickness radial deformation; (**d**) 10 mm wall thickness radial stress; (**e**) 15 mm wall thickness radial stress; (**f**) 20 mm wall thickness radial stress.

To further confirm that the N3 jump-symmetric welding sequence is the optimal welding process for pipes with three different wall thicknesses, radial deformation and stress caused by all welding processes were compared, as shown in Figure 18.

Figure 18a presents the radial deformation of the 10 mm thick pipe. It can be observed that the deformations caused by G4 and G5 processes are similar; however, combined with the radial stress results in Figure 18d, the radial welding deformation under the G5 process is significantly smaller than that under G2. Therefore, the G5 welding process is optimal for the 10 mm thick pipe.

Similarly, for the 15 mm thick pipe welding, Figure 18b shows that, except at node 7, the radial deformation caused by the G2 process is smaller at all other points compared to other welding processes. Figure 18e further illustrates that the radial stress generated by G2 is comparatively lower. Hence, the G2 welding process should be prioritized for welding the 15 mm thick pipe.

For the 20 mm thick pipe, a comprehensive comparison of radial deformation and radial stress indicates that the G4 welding process is clearly superior to the others.

In summary, based on welding simulations of circular pipes with different wall thicknesses under various welding processes, the optimal welding parameters are identified as follows: for the 10 mm thick pipe, the G5 welding process is optimal, with a current of 200 A, voltage of 24 V, and welding speed of 11.5 mm/s; for the 15 mm thick pipe, the G2 welding process is optimal, with a current of 215 A, voltage of 24.6 V, and welding speed of 10 mm/s; for the 20 mm thick pipe, the G4 welding process is optimal, with a current of 225 A, voltage of 25 V, and welding speed of 11 mm/s.

## 6. Conclusions and Future Work

In this study, finite element simulations were conducted for circular pipes with three different wall thicknesses to optimize the welding sequence and improve the welding process for shipboard thick-walled circular pipes. Based on the analysis of the welding temperature field, welding deformation, and post-weld residual stress, the following conclusions were drawn:

The use of the equal-height and equal-area method for weld seam planning and the establishment of multi-layer multi-pass welding trajectory equations, combined with the construction of a ring-shaped moving double-ellipsoid heat source model, ensured the accuracy of the welding simulation.

The segmented welding simulation of thick-walled circular pipes not only verified the correctness of welding simulation based on the double-ellipsoid heat source model but also demonstrated that the optimal N3 jump-symmetric welding sequence effectively suppressed welding deformation and residual stress, thereby achieving optimal welding quality.

Welding process improvements under the optimal jump-symmetric welding sequence revealed that as pipe wall thickness increases, welding current must also be appropriately increased. For 10 mm wall thickness: optimal process—current 200 A, voltage 24 V, welding speed 11.5 mm/s; For 15 mm wall thickness: optimal process—current 215 A, voltage 24.6 V, welding speed 10 mm/s; For 20 mm wall thickness: optimal process—current 225 A, voltage 25 V, welding speed 11 mm/s.

The present study established a finite element model based on the actual welding structural dimensions used in shipyards. However, due to the large scale of the model and limitations in research time and computational resources, only circular pipes with three wall thicknesses (10 mm, 15 mm, and 20 mm) were included in the simulation analysis. The findings may therefore exhibit a degree of contingency. Future research could extend the simulation to the welding process of pipes with greater wall thicknesses, in order to systematically evaluate the influence of wall thickness variation on welding deformation and residual stress. Furthermore, upon completion of the robotic welding system design, corresponding experimental validation will be carried out. Actual welding tests will be conducted to compare and calibrate the simulation results, thereby enhancing the comprehensiveness and engineering applicability of the study.

## Figures and Tables

**Table 1:** Model parameters of a thick-walled circular pipe.

Type	Pipe Diameter /D (mm)	Pipe Length /*L* (mm)	Backing Bar Spacing /*l* (mm)	Groove Angle /*α*	Groove Gap /*b* (mm)	Root Face /*c* (mm)
Value	500	600	320	60°	1	1

**Table 2:** Q460 Steel composition table.

Mass Fractions (%)	C	Si	Mn	S	P	Cr	Mo	Ni	Cu
Q460	≤0.22	≤0.35	0.8–1.2	≤0.02	≤0.025	≤0.25	≤0.1	≤0.3	≤0.3

**Table 3:** Heat source parameters.

Parameters	*a_f_*/mm	*a_r_*/mm	*b*/mm	*c*/mm	*f*_1_	*f*_2_
Value	4	8	4	4	0.4	1.6

**Table 4:** Welding process plan for a 10 mm thick circular pipe.

Group	Welding Current /I (A)	Welding Voltage /U (V)	Welding Speed /v (mm/s)	Heat Source Parameters			
				*a_f_*/mm	*a_r_*/mm	*b*/mm	*c*/mm
G1	180	23.2	9.5	6	3	3	3
G2	185	23.4	10	7	3.5	3.5	3.5
G3	190	23.6	10.5	8	4	4	4
G4	195	23.8	11	9	4.5	4.5	4.5
G5	200	24	11.5	10	5	5	5

**Table 5:** Welding process plan for circular pipes with wall thicknesses of 15 mm and 20 mm.

Group	Welding Current /I (A)	Welding Voltage /U (V)	Welding Speed /v (mm/s)	Heat Source Parameters			
				*a_f_*/mm	*a_r_*/mm	*b*/mm	*c*/mm
G1	210	24.4	9.5	6	3	3	3
G2	215	24.6	10	7	3.5	3.5	3.5
G3	220	24.8	10.5	8	4	4	4
G4	225	25	11	9	4.5	4.5	4.5
G5	230	25.2	11.5	10	5	5	5

## Data Availability

The original contributions presented in this study are included in the article. Further inquiries can be directed to the corresponding author.
